# The role of thymus- and extrathymus-derived regulatory T cells in maternal-fetal tolerance

**DOI:** 10.3389/fimmu.2023.1109352

**Published:** 2023-02-02

**Authors:** Zhengjuan Li, Xinyuan Liang, Xiaowen Chen, Yuying Chen, Fang Wang, Shuoshi Wang, Yihong Liao, Liping Li

**Affiliations:** Department of Obstetrics, Shenzhen People’s Hospital, The Second Clinical Medical College, Jinan University, Shenzhen, China

**Keywords:** regulatory T (Treg) cells, tTreg cells, pTreg cells, iTreg cells, Foxp3, pregnancy, maternal-fetal tolerance

## Abstract

Regulatory T (Treg) cells could be divided into thymus-derived Treg (tTreg) cells and peripherally derived Treg (pTreg) cells, and *in vitro* induced Treg (iTreg) cells. To date, the functions of tTreg versus pTreg and their relative contributions to maternal-fetal immune tolerance remain insufficiently defined due to a lack of a specific marker to distinguish tTreg cells from pTreg cells. In this study, we investigated the role of thymus- and extrathymus-derived Treg cells in pregnancy tolerance using transgenic *ACT-mOVA*, *Foxp3^DTR^* and *Foxp3^GFP^* mice, and Treg cell adoptive transfer, *etc.* We found that the frequencies of Treg cells in the thymus, spleen and lymph nodes (LNs) in either syngeneically- or allogeneically-mated pregnant mice were not different from non-pregnant mice. However, percentages of blood Treg cells in pregnant mice increased at mid-gestation, and percentages of decidua Treg cells in pregnant mice increased as the pregnancy progressed compared with non-pregnant mice, and were significantly higher in allogeneic mice than those in syngeneic group. Compared with syngeneic mice, levels of CCR2 and CCR6 on blood and decidua Treg cells and CCL12 in the decidua significantly increased in allogeneic mice. A surrogate fetal antigen mOVA that was recognized by naïve T cells from *OT-IIFoxp3^GFP^* mice induced the generation of pTreg cells *in vivo*. Transfusion of thymus and spleen Treg cells significantly decreased diphtheria toxin (DT)-increased embryo resorption rates (ERRs) and IFN-γ levels in the blood and decidua. iTreg cells also decreased ERRs and IFN-γ levels in the blood and decidua to an extent lower than thymus and spleen Treg cells. In conclusion, increased blood and decidua Treg cells in pregnancy and increased ERRs in DT-treated *Foxp3^DTR^* mice suggest an important immunosuppressive role of Treg cells in pregnancy. Elevated decidua Treg cells in pregnancy could be derived from the recruitment of tTreg cells to the decidua, or from the transformation of naïve T cells in the decidua to pTreg cells. While the immune-suppression effects of thymus and spleen Treg cells are comparable, iTreg cells might play a weaker role in maternal-fetal tolerance.

## Introduction

Regulatory T (Treg) cells are a special subset of T cells with immunomodulatory function. The transcription factor forkhead box p 3 (Foxp3) is highly expressed in the nucleus of Treg cells. Foxp3 plays an important role in the differentiation and development of Treg cells and the maintenance of their immunosuppressive function (1). The important role of Foxp3 in Treg cell function was first demonstrated by Scurfy mice with Foxp3 gene mutation that led to absence of Treg cells, proliferation of T cells in multiple organs, and death of most animals 3-5 weeks after birth (2). Similarly, it has been demonstrated that human Foxp3 gene mutation causes deletion of Treg cells and autoimmune immune disorders, leading to polyendocrine adenosis, intestinal ptosis and X-linked (IPEX) syndrome (3). Foxp3^+^ Treg cells not only regulate autoimmune diseases and maintain the stability of the internal immune environment (4), but also play an important immunosuppressive function in allergic reactions (5) and organ transplantation (6).

Treg cells are divided into thymus-derived Treg (tTreg) cells and peripherally derived Treg (pTreg) cells *in vivo* according to their sources (7). Foxp3^+^CD4^+^ tTreg cells are developed from CD4^+^CD25^+^ T cells in the thymus, while pTreg cells are mainly induced from naïve CD4^+^ T cells after they are stimulated by antigens in peripheral lymphoid organs (8). Generation of pTreg cells requires the activation of T cell receptor (TCR), and cytokines interleukin-2 (IL-2) and transforming growth factor-β (TGF-β) (8). Both tTreg cells and pTreg cells express Foxp3, and have immunosuppressive function (9). tTreg cells inhibit autoantigens and play an important role in inhibiting the body’s autoimmune responses (10). pTreg cells target exogenous antigens in the environment, air and food, and play an important role in mucosal immune tolerance and the stability of the internal immune environment (10). In addition, induced Treg (iTreg) cells can be generated *in vitro* from naïve T cells in the presence of TCR activation and cytokines (11).

tTreg cells and pTreg cells are different in gene expression, phenotypic stability, and epigenetic regulation (12). The proportion of tTreg cells and pTreg cells in the total Treg cell pool of the body, their specific functions, and how to distinguish them are not yet clear. It has been proposed that tTreg cells and pTreg cells could be distinguished by Helios since it has been reported that Helios mainly expresses on tTreg cells rather than pTreg cells (12). However, subsequent study has shown that pTreg cells also express Helios (13). Human tTreg cells also include Helios^-^ subset (14). Neuropinlin-1 has been also thought to be mainly expressed on tTreg cells rather than pTreg cells, and it has been later confirmed that Neuropinlin-1 could not distinguish tTreg cells from pTreg cells (15).

A large number of studies have shown that Treg cells may play an important role in maternal-fetal tolerance and pregnancy maintenance. Treg cells have been found to accumulate in the draining lymph nodes (LNs) of the uteri of pregnant mice and express memory and effector markers (16). Conditional removal of Treg cells from allogeneically-mated pregnant mice resulted in reduced reproductive efficiency of mice, while conditional removal of Treg cells from syngeneically-mated pregnant mice resulted in defective embryo implantation and reduced fetal size, which could be alleviated by adoptive transfer of Treg cells (17).

It has been reported that there are more inhibitory Treg cells in human decidua in early pregnancy than in peripheral blood (18). In addition, Treg cells at the maternal-fetal interface of patients with unexplained recurrent abortion were significantly reduced compared with normal pregnant women (19). Santner-nanan et al. (20) found that the number of Treg cells and the Treg/Th17 ratio in the peripheral blood of patients with preeclampsia significantly decreased compared with normal pregnant women.

Compared with syngeneically-mated pregnant mice, Treg cells in peripheral lymphoid organs but not thymus significantly increased in allogeneically-mated pregnant mice, indicating that the increase of Treg cells during pregnancy is fetal antigen specific (21). However, Aluvihare et al. (22) found that systemic amplification of Treg cells during gestation was not dependent on the stimulation of fetal antigen, but might be driven by pregnancy itself. In addition, removal of Treg cells by diphtheria toxin (DT) in both syngeneically-mated and allogeneically-mated *Foxp3^DTR^* transgenic mice resulted in embryo implantation deficiency, effector T cell infiltration, uterine inflammation and fibrosis, indicating that Treg cell-mediated pregnancy tolerance does not necessarily depend on fetus-specific antigen (23).

It has been demonstrated that Foxp3 enhancer conserved non-coding sequence 1 (CNS1) plays an important role in the generation of pTreg cells but not tTreg cells (24). Treg cells in the decidua of allogeneically-mated CNS1^-/-^ pregnant mice significantly reduced, but embryo absorption rate increased, suggesting that fetal antigen-specific pTreg cells play a major role in pregnancy tolerance (25).

To date, the functions of tTreg versus pTreg and their relative contributions to maternal-fetal immune tolerance remain insufficiently defined due to a lack of a specific marker to distinguish tTreg cells from pTreg cells. In this study, we investigated the role of thymus- and extrathymus-derived Treg cells in pregnancy tolerance using transgenic *ACT-mOVA*, *Foxp3^DTR^* and *Foxp3^GFP^* mice, and Treg cell adoptive transfer, *etc.* We found that decidua and blood Treg cells significantly increased during pregnancy; elevated decidua Treg cells in pregnancy could stem from the transformation of naïve T cells, or from the recruitment of tTreg; while the immune-suppressive effects of Treg cells in the thymus and spleen were comparable, *in vitro* generated iTreg cells might also played an important but weaker role in maternal-fetal tolerance.

## Materials and methods

### Animals

C57BL/6 and BALB/c mice were purchased from the Experimental Animal Center of Zhongshan University (Guangzhou, China). Transgenic *Foxp3^DTR^* mice, *Act-mOVA* mice and *OT-II* mice on a C57BL/6 background were purchased from The Jackson Laboratory (Bar Harbor, ME, USA). *Foxp3^GFP^* mice on a C57BL/6 background were kindly provided by Professor Zhexiong Lian (South China University of Technology, Guangzhou, China). Mice were bred in the Laboratory Animal Center of Shenzhen People’s Hospital (Shenzhen, China) in pathogen-free conditions. Syngeneic C57BL/6 × C57BL/6 and allogeneic C57BL/6 × BALB/c mating combinations were established. Each female mouse at 10-12 weeks of age was cocaged with one male. The point at which a vaginal plug was detected was designated as embryonic day 0.5 (E0.5). Mice were sacrificed at different gestational age to investigate proportions of Treg cells in the thymi, peripheral blood, spleens, LNs or deciduae. Non-pregnant mice were set up as negative controls.

### Induction of pTreg cells *in vivo*

*Foxp3^GFP^* females were mated to *OT-II* males to generate *OT-IIFoxp3^GFP^* mice. Spleen CD4^+^CD62L^+^GFP^-^ naïve T cells were isolated from female *Foxp3^GFP^* or *OT-IIFoxp3^GFP^* mice. Female C57BL/6 mice were mated to male *Act-mOVA* mice to generate fetal antigen mOVA that can be recognized by CD4^+^ cells from *OT-II* mice (26). Spleen CD4^+^CD62L^+^GFP^-^ naïve T cells (2 × 10^6^ cells) from female *Foxp3^GFP^* mice or *OT-IIFoxp3^GFP^* mice were injected into the tail veins of pregnant C57BL/6 mice mated to *Act-mOVA* mice on E9.5. Pregnant mice were sacrificed on E13.5, and CD4^+^GFP^+^ Treg cells in the peripheral blood, spleens and deciduae were analyzed using flow cytometry.

### Treg cell depletion and adoptive transfer of Treg cells

For Treg cell depletion, syngeneic pregnant *Foxp3^DTR^* mice were injected i.p. with DT (Sigma, Saint Louis, MO, USA) at a dosage of 60 µg/kg of body weight on E9.5, E10.5 and E11.5. Control mice received phosphatebuffered saline (PBS). For Treg cell reconstitution, 2 × 10^5^ purified Treg cells from the thymi and spleens of C57BL/6 mice, or *in vitro* cultured iTreg cells were injected into the tail veins of *Foxp3^DTR^* mice 2 h before DT injection. Pregnant mice were sacrificed on E13.5. At this stage of gestation, feto-placental units undergoing resorption can be clearly distinguished from their viable counterparts on the basis of size and the presence of extensive tissue wasting and hemorrhage. The number of total embryos, resorbed and viable embryos per mouse was counted. The embryo resorption rate (ERR) was calculated as: ERR (%) = number of resorbed embryos/number of total embryos × 100. Treg cells in the thymi, peripheral blood, spleens and deciduae were analyzed using flow cytometry. Deciduae were collected for calculating Th1 and Th17 cell percentages using flow cytometry.

### Isolation of thymus, spleen or LN mononuclear cells

Thymi, spleens or LNs including cervical, axillary, mesenteric and inguinal LNs were collected and mashed through a 40 μm cell strainer (Thermo Fisher Scientific, Waltham, MA, USA) to obtain single-cell suspensions. Mononuclear cells were then enriched by lysing red cells using a red cell lysis buffer (Biolegend, San Diego, CA, USA).

### Isolation of peripheral blood mononuclear cells

Peripheral blood samples were collected from the vena orbitalis, heparinized, and purified by centrifugation on a Ficoll-Hypaque Premium (GE Healthcare, Pittsburgh, PA, USA).

### Isolation of decidua mononuclear cells

Uterine tissues were collected, washed and cut into small pieces and digested with 1 mg/ml Dispase II (Roche, Basel, Switzerland) and 0.1 mg/ml DNase I (Roche) at 37°C for about 20 min in a shaking water bath. When single or clumps of cells were observed under the microscope, the released cells were separated from undigested tissue pieces by filtering through a 40 µm cell strainer. Mononuclear cells were purified over the Ficoll-Paque Premium by centrifugation at 400 g for 30 min at 20°C.

### Isolation of spleen CD4^+^CD62L^+^ naïve T cells

Naïve T cells were isolated from the spleens of *Foxp3^GFP^* mice using a mouse CD4^+^CD62L^+^ naïve T cell isolation kit (Miltenyi Biotec, Bergisch Gladbach, Germany) according to the manufacturer’s instructions. In brief, spleen mononuclear cells were labeled with a CD4^+^ T Cell Biotin-Antibody Cocktail, washed and incubated with anti-biotin microbeads. Non-CD4^+^ T cells were then magnetically depleted using the midiMACS separator. CD4^+^ T cells were collected and incubated with anti-CD62L microbeads. CD4^+^CD62L^+^ naïve T cells were sorted using the miniMACS separator. The purity of isolated naïve T cells were detected using flow cytometry.

### Isolation of thymus and spleen CD4^+^GFP^+^ Treg cells

Thymus and spleen mononuclear cells of *Foxp3^GFP^* mice on E12.5 were isolated. CD4^+^ cells were enriched using a mouse CD4^+^ T cell isolation kit (Miltenyi Biotec) according to the manufacturer’s instructions, and CD4^+^GFP^+^ Treg cells were sorted by a FACSAria cell sorter (Becton Dickinson, Franklin Lakes, NJ, USA). In brief, the single-cell suspensions were labeled with a CD4^+^ T Cell Biotin-Antibody Cocktail, washed and incubated with anti-biotin microbeads. Non-CD4^+^ T cells were then magnetically depleted using the midiMACS separator. Subsequently, enriched CD4^+^ T cells were collected, and CD4^+^GFP^+^ Treg cells were sorted using the FACS-Aria cell sorter. The purity of isolated CD4^+^GFP^+^ Treg cells were analyzed using flow cytometry.

### *In vitro* generation of spleen iTreg cells

Spleen CD4^+^CD62L^+^GFP^-^ naïve T cells of *Foxp3^GFP^* mice were isolated. The cells (1 × 10^6^/ml) were then cultured in the presence of 2.5 μg/ml anti-CD3 Ab (Biolegend), 1 μg/ml anti-CD28 Ab (Biolegend), 100 U/ml IL-2 (R&D Systems, Minneapolis, MN, USA), and 10 ng/ml TGF-β (R&D Systems) for 96 h *in vitro*. The ratio of CD4^+^GFP^+^ iTreg cells was detected using flow cytometry, and the iTreg cells were sorted for later experiments.

### Flow cytometry

For Treg cell analysis, cells were incubated with fluorescence-conjugated Abs against CD45, CD3, CD4 and CD8 for 30 min at 4°C, and then washed and fixed in a fixation buffer. Cells were re-suspended in a permeabilization wash buffer and incubated with a fluorescence-conjugated Ab against Foxp3. Isotype controls were established using matched fluorescence-labeled isotype control Abs to account for nonspecific staining. Immunostained cells were analyzed on a FACS Fortessa flow cytometer (BD Biosciences, San Jose, CA, USA) using the FlowJo software (Ashland, OR, USA). The percentages of Foxp3^+^ cells in the CD45^+^CD3^+^CD4^+^CD8^-^ cell population were analyzed.

For *Foxp3^GFP^* mice, cells were incubated with aforementioned fluorescently labeled Abs against CD45, CD3, CD4 and CD8 for 30 min at 4°C. Cells were washed and fixed in a fixation buffer. The percentage of GFP^+^ cells in the CD4^+^GFP^+^ cell population was analyzed. In selected experiments, cells were incubated with fluorescently labeled Abs against CD45, CD3, CD4 and CD8, CCR2 and CCR6. The expression of CCR2 and CCR6 on CD45^+^CD3^+^CD4^+^CD8^-^GFP^+^ cells was detected. For naïve T cell analysis, cells were mixed with fluorescently labeled Abs against CD45, CD3, CD4 and CD8, and CD62L for 30 min at 4°C. The percentages of CD45^+^CD3^+^CD4^+^CD62L^+^ cells in CD45^+^CD3^+^CD4^+^CD8^-^ cells were analyzed.

For analysing intracellular factors, cells were incubated with fluoresence-conjugated Abs against CD45, CD3, CD4 and CD8 for 30 min at 4°C. Cells were washed, fixed, re-suspended in a permeabilization wash buffer, and incubated with fluorescence-conjugated Abs against IFN-γ and IL-17. Percentages of IFN-γ^+^ and IL-17^+^ cells in the CD45^+^CD3^+^CD4^+^CD8^-^ cell population were analyzed. Fluorescence-conjugated Abs, matched fluorescence-labeled isotype control Abs, fixation buffer, and permeabilization wash buffer were all purchased from BioLegend. Flow cytometry Abs were summarized in **Table 1**.

**Table 1:** Ab information.

Ab	Company
APC/Cyanine7anti-mouse CD45 Ab	Biolegend
Brilliant Violet 421™ anti-mouse CD3Ab	Biolegend
PerCP/Cyanine5.5 anti-mouse CD4 Ab	Biolegend
Brilliant Violet 785™ anti-mouse CD8 Ab	Biolegend
Alexa Fluor^®^ 647 anti-mouse FOXP3 Ab	Biolegend
APC anti-mouse CCR2 Ab	Biolegend
PE/Cyanine7 anti-mouse CCR6 Ab	Biolegend
FITC anti-mouse CD62L Ab	Biolegend
APC anti-mouse IFN-γ Ab	Biolegend
PE anti-mouse IL-17A Ab	Biolegend
APC/Cyanine7 Rat IgG2b, κ Isotype Ctrl Ab	Biolegend
Brilliant Violet 421™ Armenian Hamster IgG Isotype Ctrl Ab	Biolegend
PerCP/Cyanine5.5 Rat IgG2b, κ Isotype Ctrl Ab	Biolegend
Brilliant Violet 785™ Rat IgG2a, κ Isotype Ctrl Ab	Biolegend
Alexa Fluor^®^ 647 Rat IgG2b, κ Isotype Ctrl Ab	Biolegend
APC Rat IgG2b, κ Isotype Ctrl Ab	Biolegend
PE/Cyanine7 Rat IgG2b, κ Isotype Ctrl Ab	Biolegend
FITC Rat IgG2a, κ Isotype Ctrl Ab	Biolegend
APC Rat IgG1, κ Isotype Ctrl Ab	Biolegend
PE Rat IgG1, κ Isotype Ctrl Ab	Biolegend
Murine CCL2 affinity purified polyclonal Ab	Abcam
Murine CCL7 affinity purified polyclonal Ab	R&D Systems
Murine CCL12 affinity purified polyclonal Ab	R&D Systems
Murine CCL20 affinity purified polyclonal Ab	Abcam
Horseradish peroxidase (HRP)-coupled goat-anti-mouse secondary Ab IgG (H+L)	Thermo Fisher Scientific

### mRNA levels of chemokines in the decidua by real time (RT)-PCR

The uteri of the non-pregnant mice, syngeneic and allogeneic pregnant mice on E13.5 were quickly removed and placed in liquid nitrogen and transferred to the -80°C refrigerator for later use. Total RNA was extracted from the uteri using Trizol extraction, and the content and purity of total RNA were determined by spectrophotometry. cDNA was obtained according to the instructions of the PrimeScript™ RT Reagent Kit with gDNA Eraser (Perfect Real Time) (TaKaRa, Beijing, China). The mRNA levels of *ccl2, ccl7, ccl12,ccl17, ccl19, ccl20, ccl21, cxcl9, cxcl10* and *cxcl11* in the deciduae were detected using PowerUp™ SYBR™ Green Master Mix (Applied Biosystems, Foster City, CA, US) and QuantStudio fluorescent quantitative RT-PCR system (Thermo Fisher Scientific) with β-actin as the internal reference. The method of 2^⁃△△Ct^ relative quantitation was used to compare the difference of target mRNA expression between groups. The primers of the chemokines were bought from Shanghai Shenggong Biological Company (Shanghai, China) and shown in the **Table 2**.

**Table 2:** The primers for chemokines.

Genes	Upstream primers	Downstream primers
*Ccl2*	5’-TTAAAAACCTGGATCGGAACCAA-3’	5’-GCATTAGCTTCAGATTTACGGGT-3’
*Ccl7*	5’-AAGTGGGTCGAGGAGGCTAT-3’	5’-AAGTGGGTCGAGGAGGCTAT-3’
*Ccl12*	5’-ATTTCCACACTTCTATGCCTCCT-3’	5’-ATCCAGTATGGTCCTGAAGATCA-3’
*Ccl17*	5’-TACCATGAGGTCACTTCAGATGC-3’	5’-GCACTCTCGGCCTACATTGG-3’
*Ccl19*	5’-GGGGTGCTAATGATGCGGAA-3’	5’-CCTTAGTGTGGTGAACACAACA-3’
*Ccl20*	5’-GCCTCTCGTACATACAGACGC-3’	5’-CCAGTTCTGCTTTGGATCAGC-3’
*Ccl21*	5’-GTGCAGAAGTTGATGCGACG-3’	5’-CCCAGCTTGAAGTTCGTGGA-3’
*Cxcl9*	5’-GGAGTTCGAGGAACCCTAGTG-3’	5’-GGGATTTGTAGTGGATCGTGC-3’
*Cxcl10*	5’-CCAAGTGCTGCCGTCATTTTC-3’	5’-GGCTCGCAGGGATGATTTCAA-3’
*Cxcl11*	5’-GGCTTCCTTATGTTCAAACAGGG-3’	5’-GGCTTCCTTATGTTCAAACAGGG-3’
β*-actin*	5’-GGCTGTATTCCCCTCCATCG-3’	5’-CCAGTTGGTAACAATGCCATGT-3’

### Protein levels of chemokines in the decidua by western blot

The uteri of the non-pregnant mice, syngeneic and allogeneic pregnant mice on E13.5 were collected and stored at -80°C. Tissue protein was extracted, and the bicinchoninic acid (BCA) Protein Quantification Kit (Thermo Fisher Scientific) was used to detect protein expression levels. The prestain protein marker and protein samples to be tested were loaded in the glue and electrophoresed, and then transferred to the PVDF membrane, and sealed by 5% skimmed milk. The membrane was then incubated with anti-mouse CCL2, CCL7, CCL12 and CCL20 Abs, washed and incubated with horseradish peroxidase (HRP)-coupled goat-anti-mouse secondary Ab IgG (H+L). The membrane was then washed, and hypersensitive chemiluminescence substrate (Thermo Fisher Scientific) was added for color exposure. Image J software (NationalnInstitutes of Health, Bethesda, MD, USA) was used to detect the gray value of the strip. The expression of target proteins CCL2, CCL7, CCL12 and CCL20 were analyzed semi-quantitatively using GAPDH as the internal reference protein. Western blot Abs were summarized in **Table 1**.

### Chemotaxis experiments

Isolated thymus CD4^+^GFP^+^ Treg cells (1 × 10^5^) were seeded in the upper chamber of a 24-well plate (5-μm pore; Sigma). A volume of 600 μl recombinated chemokines including 20 ng/ml CCL2, 500 ng/ml CCL7, 50 ng/ml CCL12 and 10 ng/ml CCL20 were loaded in the lower chamber, respectively. Basal culture medium in the lower chamber served as a negative control. After 24 h, cells in the lower chamber were collected. The numbers of CD4^+^GFP^+^ cells were calculated using flow cytometry. Migration index was expressed as fold change of the numbers of CD4^+^GFP^+^ cells relative to those isolated from basal culture medium controls. The assay was carried out in triplicate and repeated three times independently.

### Statistical analysis

All statistical analyses were performed using SPSS 19.0 software (Chicago, IL, USA). Data were analyzed by one-way ANOVA with Bonferroni test among three or more groups, or independent Student’s *t*-test between two groups. Results were given as mean ± SD. A *p* value of < 0.05 was considered statistically significant between analyzed groups.

## Results

### Percentages of thymus, blood, spleen and LN Treg cells in syngeneic and allogeneic pregnancies

**Figure 1:**
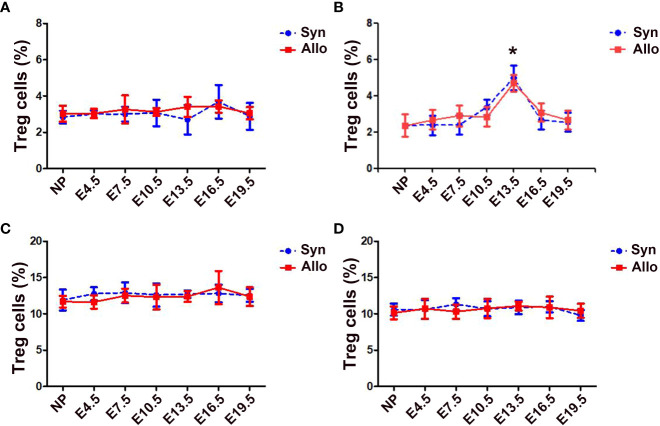
Percentages of thymus, blood, spleen and LN Treg cells in syngeneic and allogeneic pregnancies. A graphical summary of the percentages of thymus **(A)**, blood **(B)**, spleen **(C)** and LN **(D)** Treg cells is presented. Data are presented as mean ± SD (n = 6). ^*^ *p* < 0.01 vs the non-pregnant mice. NP, non-pregnant group; Syn, syngeneic pregnant group; Allo, allogeneic pregnant group.

The percentages of thymus, spleen and LN CD4^+^Foxp3^+^ Treg cells in CD4^+^ T cells neither differ significantly among pregnant and non-pregnant groups, nor differ significantly between syngeneic and allogeneic mice from E4.5 to E19.5 (**Figures 1A, C, D**). However, as shown in **Figure 1B**, the proportions of blood CD4^+^Foxp3^+^ Treg cells in CD4^+^ T cells significantly increased in both syngeneic and allogeneic mice on E13.5 as compared with non-pregnant mice (*p* < 0.01 for both comparisons), while proportions of blood Treg cells at different gestational ages did not differ between syngeneic and heterogeneic pregnant mice.

### Percentages of decidua Treg cells in syngeneic and allogeneic pregnancies

**Figure 2:**
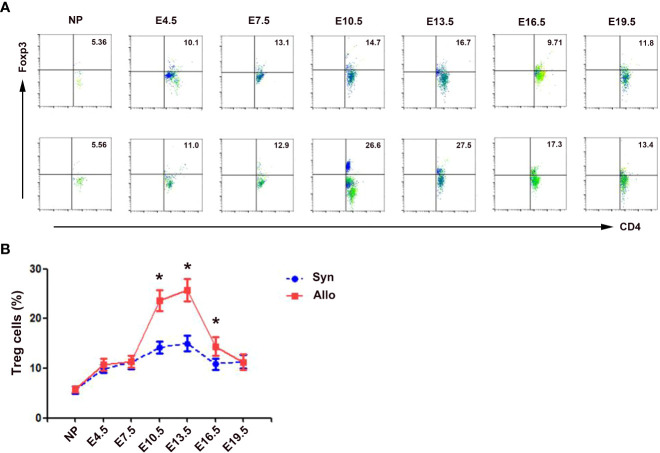
Percentages of decidua Treg cells in syngeneic and allogeneic pregnancies. **(A)** One representative experiment of the percentages of decidua Treg cells in syngeneic mice (upper) and allogeneic mice (lower) is shown. Numbers indicate the percentages of Foxp3^+^CD4^+^ cells in CD4^+^ T cells (%). **(B)** A graphical summary of the percentages of decidua Treg cells is presented. Data are presented as mean ± SD (n = 6). ^*^ *p* < 0.01 vs the syngeneic mice. NP, non-pregnant group; Syn, syngeneic pregnant group; Allo, allogeneic pregnant group.

As shown in **Figure 2**, the percentages of decidua CD4^+^Foxp3^+^ Treg cells in both syngeneic and allogeneic mice significantly increased as the pregnancy progressed, peaked on E13.5, and then decreased till E19.5. The percentages of decidua Treg cells in allogeneic mice on E10.5, E13.5 and E16.5 were significantly higher than those in syngeneic mice (*p* < 0.01, respectively).

### mRNA and protein levels of chemokines in the deciduae of syngeneic and allogeneic mice

**Figure 3:**
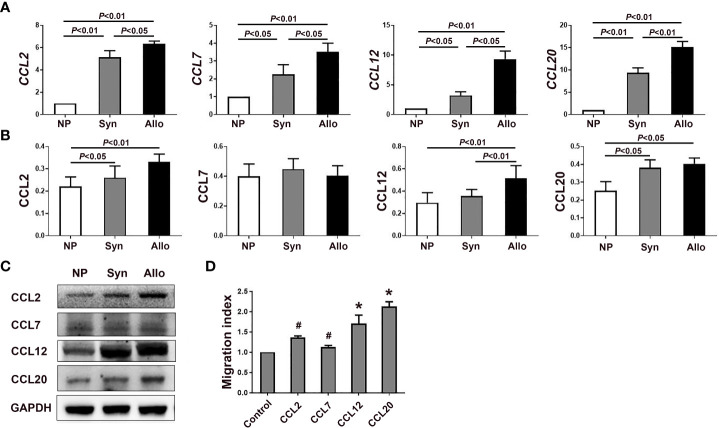
mRNA and protein levels of chemokines in the deciduae of syngeneic and allogeneic mice. **(A)** Levels of *CCL2*, *CCL7*, *CCL12* and *CCL20* mRNA in the deciduae of syngeneic and allogeneic mice are shown (n = 6). **(B)** Levels of proteins CCL2, CCL7, CCL12 and CCL20 in the deciduae of syngeneic and allogeneic mice are shown. **(C)** One representative western blot experiment of the protein levels of CCL2, CCL7, CCL12 and CCL20 is shown. **(D)** Chemotactic effects of chemokines CCL2, CCL7, CCL12 and CCL20 on thymus Treg cells. Data are presented as mean ± SD (n = 6). ^*^ *p* < 0.01, ^#^ *p* < 0.05 vs the control group.

As shown in **Figure 3A**, compared with non-pregnant group, levels of *CCL2*, *CCL7*, *CCL12* and *CCL20* mRNA in the deciduae significantly increased in both syngeneic mice (*p* < 0.01, *p* < 0.05, *p* < 0.05 and *p* < 0.01) and in allogeneic mice (*p* < 0.01, respectively). In addition, levels of *CCL2*, *CCL7*, *CCL12* and *CCL20* mRNA in the deciduae of allogeneic mice were significantly up-regulated compared with those of syngeneic mice (*p* < 0.05, *p* < 0.05, *p* < 0.05 and *p* < 0.01). However, levels of *CCL17*, *CCL19*, *CCL21*, *CXCL9*, *CXCL10* and *CXCL11* mRNA in the deciduae of pregnant mice did not differ significantly from non-pregnant mice (data not shown).

Since mRNA levels of *CCL2*, *CCL7*, *CCL12* and *CCL20* significantly increased in the deciduae of pregnant mice compared with non-pregnant mice, we analyzed protein levels of those chemokines in the decidua. As shown in **Figures 3B, C**, protein levels of CCL2, CCL12 and CCL20 in the deciduae of syngeneic mice (*p* < 0.05, *p* < 0.01 and *p* < 0.05) and allogeneic mice (*p* < 0.01, *p* < 0.01 and *p* < 0.05) were significantly higher than those of non-pregnant mice. In addition, the level of CCL12 in the deciduae of allogeneic mice significantly increased compared with that of syngeneic mice (*p* < 0.01). However, we did not find significant differences in CCL7 levels among the three groups (**Figures 3B, C**).

### Chemotactic effects of chemokines CCL2, CCL12 and CCL20 on thymus Treg cells

In the case the mRNA levels of *CCL2*, *CCL7*, *CCL12* and *CCL20* in the deciduae significantly increased, we wondered whether those chemokines can attract thymus Treg cells into the deciduae by chemotactic experiments *in vitro*. As shown in **Figure 3D**, the migration indexes of CCL2, CCL7, CCL12 and CCL20 groups were all significantly increased compared with the control group without chemokines (*p* < 0.05, *p* < 0.01 and *p* < 0.01).

### Expression of chemokine receptors CCR2 and CCR6 on thymus and blood Treg cells between syngeneic and allogeneic mice

**Figure 4:**
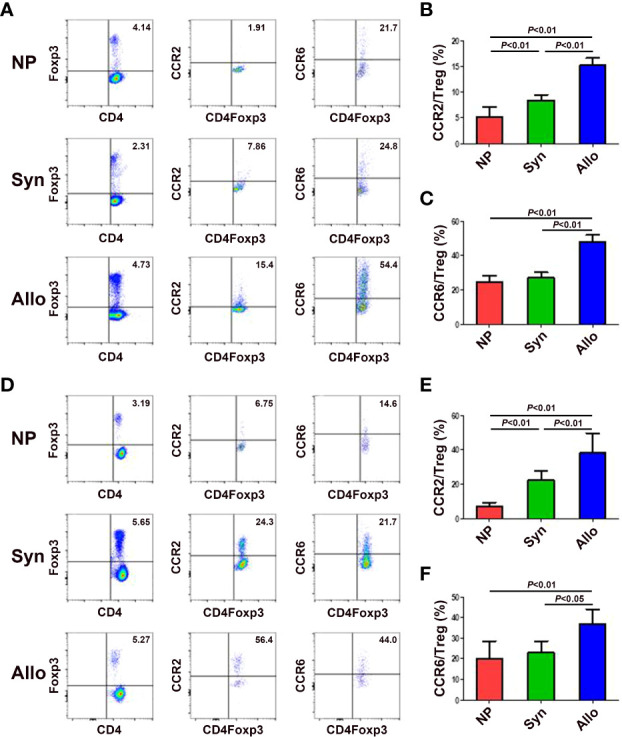
Expression of chemokine receptors CCR2 and CCR6 on thymus and blood Treg cells between syngeneic and allogeneic mice. **(A)** Representative flow cytometry experiments of the levels of CCR2 and CCR6 on thymus Treg cells are shown. Levels of CCR2 **(B)** and CCR6 **(C)** on thymus Treg cells are shown. **(D)** Representative flow cytometry experiments of the levels of CCR2 and CCR6 on peripheral blood Treg cells are shown. Levels of CCR2 **(E)** and CCR6 **(F)** on peripheral blood Treg cells are shown. Data are presented as mean ± SD (n = 6). NP, non-pregnant group; Syn, syngeneic pregnant group; Allo, allogeneic pregnant group.

Since mRNA and protein levels of CCL2, CCL12 and CCL20 in the decidua significantly increased in pregnant mice, we wanted to know levels of the receptors CCR2 and CCR6 of those chemokines on thymus and blood Treg cells. As shown in **Figures 4A-C**, compared with non-pregnant mice, while the expression of CCR2 on thymus Treg cells significantly up-regulated in syngeneic and allogeneic mice (*p* < 0.01 for both comparisons), the expression of CCR6 on thymus Treg cells significantly up-regulated in allogeneic mice (*p* < 0.01). In addition, the expression of CCR2 and CCR6 on thymus Treg cells significantly increased in allogeneic mice as compared to syngeneic mice (*p* < 0.01 for both comparisons). Similarly, as shown in **Figures 4D-F**, compared with non-pregnant mice, while the expression of CCR2 on peripheral blood Treg cells significantly up-regulated in syngeneic and allogeneic mice (*p* < 0.01 for both comparisons), the expression of CCR6 on peripheral blood Treg cells significantly up-regulated in allogeneic mice (*p* < 0.01). Besides, the expression of CCR2 (*p* < 0.01) and CCR6 (*p* < 0.05) on peripheral blood Treg cells significantly increased in allogeneic mice as compared to syngeneic mice.

### Fetal antigen induced pTreg cell generation *in vivo*

**Figure 5:**
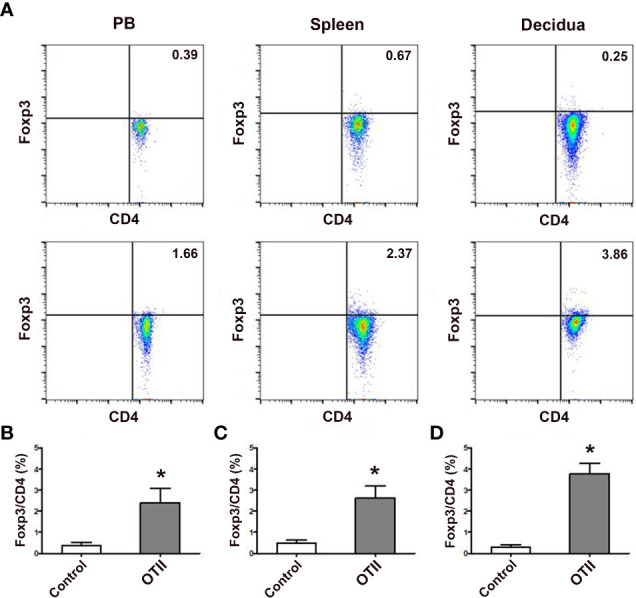
Fetal antigen induced pTreg cell generation *in vivo*. **(A)** Representative flow cytometry experiments of *in vivo* generated CD4^+^GFP^+^ Treg cells from naïve T cells of female *Foxp3^GFP^* mice (upper row) or *OT-IIFoxp3^GFP^* mice (lower row) in the peripheral blood, spleens and deciduae of pregnant C57BL/6 mice mated to *Act-mOVA* mice are shown. Levels of *in vivo* generated CD4^+^GFP^+^ Treg cells in the peripheral blood **(B)**, spleens **(C)** and deciduae **(D)** of pregnant C57BL/6 mice mated to Act-mOVA mice are shown. Data are presented as mean ± SD (n = 6). ^*^ *p* < 0.01 vs the control group.

In order to investigate the role of fetal antigen-specific pTreg cells in maternal-fetal tolerance, we first investigated whether pTreg could be induced from fetal antigen-specific naïve T cells using transgenic *OT-II* mice and fetal surrogate antigen mOVA. We demonstrated that pTreg cells were induced *in vivo* from mOVA-specific CD4^+^CD62L^+^GFP^-^ naïve T cells, since transfusion of CD4^+^CD62L^+^GFP^-^ naïve T cells that from *OT-IIFoxp3^GFP^* mice induced more CD4^+^GFP^+^ pTreg cells in the peripheral blood, spleens and deciduae of recipient mice than transfusion of naïve T cells from *Foxp3^GFP^* mice (*p* < 0.01, respectively) (**Figure 5**).

### Adoptive transfer of Treg cells inhibits DT-induced pregnancy loss in *Foxp3^GFP^* mice

**Figure 6:**
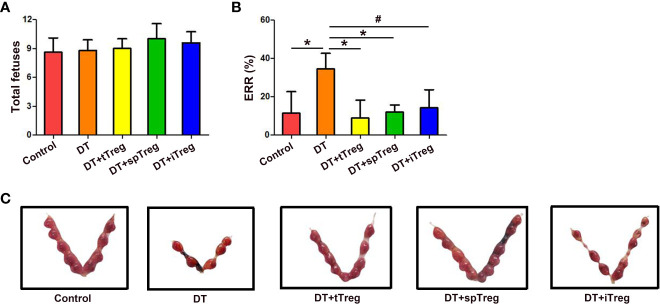
Adoptive transfer of Treg cells inhibits DT-induced pregnancy loss in *Foxp3^GFP^* mice. The number of total fetuses **(A)** and ERRs **(B)** were calculated. Data are presented as mean ± SD (n = 8). ^*^ *p* < 0.01, ^#^ *p* < 0.05. **(C)** A representative macroscopic view of fetuses from mice that received vehicle, DT, DT plus tTreg cells, DT plus spTreg cells, or DT plus iTreg cells is shown. spTreg, spleen Treg cells.

In order to investigate the role of Treg cells in pregnancy, pregnant *Foxp3^DTR^* mice were i.p. injected with DT to deplete Treg cells to set a murine inflammatory abortion model. Our results showed that 3 consecutive injections of DT at the dosage of 60 μg/kg weight almost completely deplete body Treg cells as demonstrated by nearly no Treg cells in the thymi, blood, LNs, spleens and deciduae without obvious maternal morbidity or mortality. Treatment with DT did not change total fetuses, but markedly increased the ERR in *Foxp3^DTR^* mice (*p* < 0.01) (**Figure 6**), suggesting an important role of Treg cells in maternal-fetal tolerance.

Meanwhile, we investigated whether thymus- or extrathymus-derived Treg cells play a role in pregnancy tolerance by adoptive transfer of Treg cells from different sources into DT-injected *Foxp3^DTR^* mice. As shown in **Figure 6A**, total fetuses did not differ among groups. Adoptive transfer of thymus (*p* < 0.01) or spleen CD4^+^GFP^+^ Treg cells (*p* < 0.01), or *in vitro* generated iTreg cells (*p* < 0.05) into DT-treated *Foxp3^DTR^* mice significantly decreased ERRs compared with DT-treated *Foxp3^DTR^* mice, which did not differ from those of vehicle-treated control mice (**Figures 6B, C**).

### Effects of adoptive transfer of Treg cells on peripheral blood and decidua Th1 and Th17 cells in DT-treated *Foxp3^DTR^* mice

**Figure 7:**
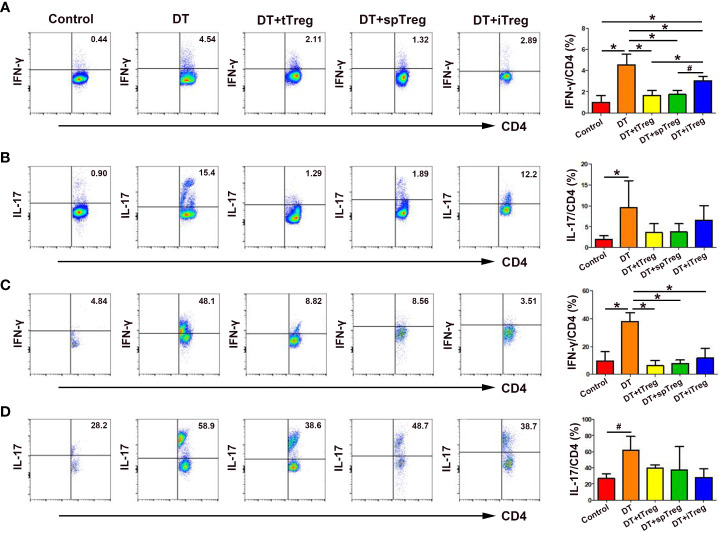
Effects of adoptive transfer of Treg cells on peripheral blood and decidua Th1 and Th17 cells in DT-treated *Foxp3^DTR^* mice. Representative flow cytometry experiments and levels of IFN-γ **(A)** and IL-17 **(B)** on peripheral blood CD4^+^ T cells are shown. Representative flow cytometry experiments and levels of IFN-γ **(C)** and IL-17 **(D)** on decidua CD4^+^ T cells are presented. Data are presented as mean ± SD (n = 6). ^*^ *p* < 0.01, ^#^ *p* < 0.05.

Since Th1 and Th17 cells play an important role in pregnancy loss (27), we wondered whether the immune tolerant effects by adoptive transfer of Treg cells were through inhibiting Th1 or Th17 cells. We found that treatment with DT significantly increased the percentages of peripheral blood IFN-γ^+^ cells and IL-17^+^ cells in CD45^+^CD3^+^CD4^+^ cells compared with controls (*p* < 0.01 for both comparisons, **Figures 7A, B**), indicating that deficiency of Treg cells increases systemic Th1 and Th17 cells. Compared with DT-treated *Foxp3^DTR^* mice, adoptive transfer of thymus or spleen CD4^+^GFP^+^ Treg cells significantly decreased the percentages of peripheral blood IFN-γ^+^ cells (*p* < 0.01 for both comparisons), which did not differ from those of vehicle-treated control mice (**Figure 7A**). In addition, compared with DT-treated *Foxp3^DTR^* mice, adoptive transfer of *in vitro* generated iTreg cells significantly decreased the percentages of peripheral blood IFN-γ^+^ cells (*p* < 0.01), which were still higher than those of vehicle-treated control mice (*p* < 0.01), and DT-treated *Foxp3^DTR^* mice with adoptive transfer of thymus Treg cells (*p* < 0.01) or spleen Treg cells (*p* < 0.05, **Figure 7A**).

Similarly, treatment with DT significantly increased the percentages of decidua IFN-γ^+^ cells (*p* < 0.01) and IL-17^+^ cells (*p* < 0.05) in CD45^+^CD3^+^CD4^+^ cells compared with controls (**Figures 7C, D**), indicating that deficiency of Treg cells also increases maternal-fetal interface Th1 and Th17 cells. Compared with DT-treated *Foxp3^DTR^* mice, adoptive transfer of thymus and spleen Treg cells or *in vitro* generated iTreg cells significantly decreased the percentages of decidua IFN-γ^+^ cells (*p* < 0.01, respectively), which did not differ from those of vehicle-treated control mice (**Figure 7C**).Compared with DT-treated *Foxp3^DTR^* mice, adoptive transfer of thymus and spleen Treg cells or *in vitro* generated iTreg cells decreased the percentages of peripheral blood and decidua IL-17^+^ cells, but the differences did not reach statistical significance (**Figures 7B, D**).

## Discussion

The genome of the fetus consists of both the tissue-specific antigens and paternally-inherited antigens (28). “Self”-antigens that are highly restricted to the fetus and placenta, are promiscuously expressed by medullary thymic epithelial cells under the control of Autoimmune Regulator (Aire) (29). Treg cells that circulate in mothers during pregnancy may be comprised of Tregs that are derived from the thymus as well as those are induced in the periphery. Exogenous fetal/placental antigens, including paternally-inherited major and minor histocompatibility antigens that differ between parents (30) may elicit elevated pTreg cells in the placenta. Indeed, significantly increased decidua Treg cells in allogeneic pregnant mice compared to syngeneic mice in our study suggests that at least part of increased decidua Treg cells may be derived from locally induced pTreg in response to fetus-specific paternally-inherited antigens in allogeneic mice. Similarly, scholars have reported that exogenous fetal/placental antigens induce the generation of pTreg in the decidua (25). In addition, extravillous trophoblast cells can induce Foxp3 expression in naive CD4^+^ T cells through a contact-independent manner, suggesting that a non-antigenic stimulation may induce pTreg cell generation in the decidua (31).

Most studies in mice agree that both syngeneic and allogeneic pregnancies involve early and local expansion of Treg cells, and an earlier and higher degree of expansion occurs in allogeneic pregnancies (16, 32, 33). In addition to locally induced pTreg cells in the decidua, up-regulated decidua Treg cells may also stem from tTreg cells since blood Treg cells in both syngeneic and allogeneic mice significantly increased at mid-gestation, and decidua Treg cells in syngeneic mice also significantly increased compared to non-pregnant mice. It has been reported that exposure of endometrium to seminal fluid induces expansion of Treg cells in the preimplantation mouse uterus (34). Accordantly, we found elevated Treg cells in the decidua as early as E4.5 in our study.

Until now, it has been difficult to directly prove the role of fetus-specific Treg cells in pregnancy tolerance due to the lack of direct manipulation of fetus-specific antigen (32). In our study, female C57BL/6 mice were mated to male *Act-mOVA* mice to generate a surrogate fetal antigen mOVA, which could be recognized by naïve T cells from *OT-IIFoxp3^GFP^* mice and induce the generation of pTreg cells in blood, spleens and deciduae. Similarly, Rowe et al. (35) have demonstrated that a substitute fetal antigen 2W1S_55-66_ induces Treg cell proliferation which remain resistant to the previous fetal antigen after delivery and rapidly expand in the second pregnancy. The progeny from C57BL/6 mice and BALB/C mice expresses peptide Eα_55-66_ derived from the MHC II molecule I-ED, which can be recognized by the TCR of transgenic TEa mice, and promote the transformation of T cells of TEa mice into Treg cells in a CNS1-dependent manner (25). Our results suggest that elevated decidua Treg cells could be derived from the transformation of naïve T cells in the decidua to pTreg cells after they recognize fetal antigens inherited from the father.

Part of increased decidua Treg cells may also originate from the recruitment of tTreg cells to the decidua. Elevated mRNA and protein levels of chemokines CCL2, CCL12 and CCL20 in the decidua and increased levels of chemokine receptors CCR2 and CCR6 on thymus and peripheral blood Treg cells indicates that Treg cells in the thymus could recruit to the decidua during pregnancy. We also found elevated mRNA but unchanged protein levels of CCL7 in the decidua of pregnant mice compared with non-pregnant mice in our study. The expression of *CCL7* might be affected by various external and internal factors, including RNA interference, and showed no expression. In addition, our chemotaxis experiments have proved that chemokines CCL2, CCL12 and CCL20 do promote the migration of thymus Treg cells. Increased levels of decidual chemokines and chemokine receptors on thymus and peripheral blood Treg cells in allogeneic mice compared to syngeneic mice, suggests that paternally inherited antigens may also recruit tTreg cells to the decidua.

While pTreg cells play a role in maternal tolerance to the fetus (25), a possible role of central tolerance in pregnancy is less well understood and explored. Although CNS1-deleted transgenic mice were used to study the role of pTreg cells in pregnancy tolerance, deletion of the CNS1 region in Foxp3 did not completely abrogate Foxp3 induction (36). CNS1 deficiency in Foxp3 delays, but does not abrogate Treg cell selection (36). Therefore, we adoptively transferred of Treg cells from different sources into DT-injected *Foxp3^DTR^* mice to investigate their relative roles in maternal-fetal tolerance.

Interestingly, thymus Treg cells and spleen Treg cells that include thymus- and extrathymus-derived Tregs (12, 37) played a comparable role in decreasing DT- increased ERRs and IFN-γ levels in the blood and deciduae of *Foxp3^DTR^* mice. Unexpectedly, thymus Treg cells also had significant effects in suppressing immune activation in pregnancy. Indeed, it has been reported that pTreg cell deficiency induced by CNS1 depletion leads to a moderate increase of pregnancy loss in mice (25), indirectly indicating that tTreg cells may also play an important role in maternal-fetal tolerance.

Th17 cells are a newly discovered subset of Th cells different from Th1 and Th2 cells (38). They mainly secrete IL-17 and play an important role in pregnancy loss (27). In our study, transfer with thymus and spleen Treg cells, or iTreg cells decreased Th17 cells in DT-treated *Foxp3^DTR^* mice, but the differences did not reach statistical significance. Our data suggest that adoptive transfer of Treg cells may play a limited role in decreasing Th17 cells in DT-induced pregnancy loss model.

A number of studies have suggested that Foxp3^+^ Treg cells retain plasticity and can be “reprogrammed” into T helper cells under certain environmental conditions (39, 40). It has been demonstrated that *in vitro* induced iTreg cells have highly methylated Treg specific demethylation region (TSDR) and are prone to lose Foxp3 (41, 42). However, in our study, *in vitro* generated iTreg cells also demonstrated a role in inhibiting Treg cell deficiency-induced pregnancy loss, and blood and maternal-fetal immune activation. Since iTreg cells decreased ERRs and IFN-γ levels in the blood and decidua to an extent lower than thymus and spleen Treg cells, we consider that their effects may be weaker than those of tTreg cells and spleen Treg cells.

In conclusion, increased decidua Treg cells and DT-increased ERRs in *Foxp3^DTR^* mice suggest an immunosuppressive role of Treg cells in pregnancy. Elevated decidua Treg cells in pregnancy could be derived from the transformation of naïve T cells in the decidua to pTreg cells, or from the recruitment of tTreg cells to the decidua. The immune-suppression effects of tTreg cells and spleen Treg cells including both tTreg and pTreg cells are comparable. *In vitro* generated iTreg cells may also play a role in maternal-fetal tolerance, although the effects might be relatively weaker than those of tTreg cells and spleen Treg cells.

## Data availability statement

The original contributions presented in the study are included in the article/supplementary material. Further inquiries can be directed to the corresponding authors.

## Ethics statement

The animal study was reviewed and approved by the Animal Care and Use Committee of Jinan University.

## Author contributions

ZL, XL and XC performed experiments and drafted the manuscript. YC, FW and SW analyzed and interpreted the data. LL was responsible for the conception and design of this project. LL and YL revised the manuscript and provided overall direction. All authors contributed to the article and approved the submitted version.
